# Highly Sensitive Detection of Human Pluripotent Stem Cells by Loop-Mediated Isothermal Amplification

**DOI:** 10.1007/s12015-022-10402-3

**Published:** 2022-06-03

**Authors:** Ryota Yasui, Atsuka Matsui, Keisuke Sekine, Satoshi Okamoto, Hideki Taniguchi

**Affiliations:** 1 Department of Regenerative Medicine, Yokohama City University Graduate School of Medicine, Yokohama, Kanagawa 236-0004 Japan; 2 Fundamental Research Laboratory, Eiken Chemical Co., Ltd., Nogi, Tochigi 329-0114 Japan; 3 Biochemical Research Laboratory, Eiken Chemical Co., Ltd., Ohtawara, Tochigi 324-0036 Japan; 4 Laboratory of Cancer Cell Systems, National Cancer Center Research Institute, 5-1-1 Tsukiji, Chuo-ku, Tokyo, 104-0045 Japan; 5 Division of Regenerative Medicine, Center for Stem Cell Biology and Regenerative Medicine, The Institute of Medical Science, The University of Tokyo, 4-6-1 Shirokanedai, Minato-ku, Tokyo, 108-8639 Japan

**Keywords:** Regenerative medicine, Pluripotent stem cell, Loop-mediated isothermal amplification (LAMP), Regulatory science

## Abstract

For safe regenerative medicines, contaminated or remaining tumorigenic undifferentiated cells in cell-derived products must be rigorously assessed through sensitive assays. Although in vitro nucleic acid tests offer particularly sensitive tumorigenicity-associated assays, the human pluripotent stem cell (hPSC) detectability is partly constrained by the small input amount of RNA per test. To overcome this limitation, we developed reverse transcription loop-mediated isothermal amplification (RT-LAMP) assays that are highly gene specific and robust against interfering materials. LAMP could readily assay microgram order of input sample per test and detected an equivalent model of 0.00002% hiPSC contamination in a simple one-pot reaction. For the evaluation of cell-derived total RNA, RT-LAMP detected spiked-in hPSCs among hPSC-derived trilineage cells utilizing multiple pluripotency RNAs. We also developed multiplex RT-LAMP assays and further applied for in situ cell imaging, achieving specific co-staining of pluripotency proteins and RNAs. Our attempts uncovered the utility of RT-LAMP approaches for tumorigenicity-associated assays, supporting practical applications of regenerative medicine.

**Graphical Abstract:**

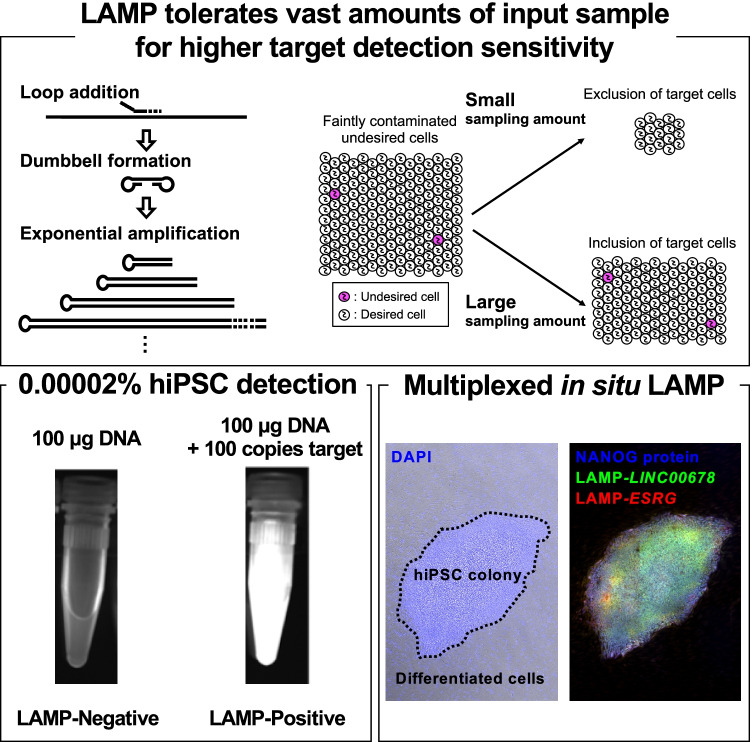

**Supplementary Information:**

The online version contains supplementary material available at 10.1007/s12015-022-10402-3.

## Introduction

Accumulating evidence from pioneering clinical trials and authorization of cell- and tissue-engineered products have demonstrated potential benefits of regenerative medicine. Thus far, a number of legally approved autologous products, (e.g., mesenchymal stem cells [MSCs], chondrocytes, antigen presenting cells, limbal stem cells, neural cells, and epidermal or cardiac cell sheets) and approved allogeneic materials (e.g., MSCs, fibroblasts, keratinocytes, hematopoietic progenitor cells, and epidermal cell sheets) are used in regenerative medicine. Among various cell sources for regenerative medicine, human pluripotent stem cells (hPSCs), which include human-induced pluripotent stem cells (hiPSCs) and human embryonic stem cells, are attractive cell therapy materials due to their massive growth capacity and unlimited differentiation capability. Currently, more than 30 clinical trials using hPSC-derived cell therapy products are ongoing worldwide [1, 2].

The safety and reliability of cell-derived materials in clinical applications requires prudent tumorigenicity risk assessments, which are mandatory and crucial especially for hPSC-derived materials, because undifferentiated hPSCs have the potential risk of teratoma formation [3, 4]. Under supportive conditions for tumor growth, hundreds of hPSCs were demonstrated to cause teratoma formation in an immunodeficient model mice [4–6]. Therefore, undifferentiated cells that stubbornly remain after the differentiation process indicate that there are considerable risks of hPSC applications. For treatments requiring a vast number of cells, such as 1 × 10^8^ or more cells [7] for heart failure [8], acute graft versus host disease [9], and liver failure treatment [10], the detection of minor undifferentiated cells in hPSC-derived end materials is extremely difficult. To overcome these tumorigenicity issues, novel technologies have been developed to remove undifferentiated cells or accumulating differentiated cells [3, 11–15]. As for in vitro detection of undifferentiated cells, culture assay [16] and polymerase chain reaction (PCR)-based nucleic acid tests quantitating pluripotency marker RNAs [17–19] have achieved sensitive undifferentiated cell detection. Nucleic acid tests can be performed in a day, and thus excel in test rapidity and throughput, whereas culture assays require days of cultivation for undifferentiated cell colony formation.

For some methods of undifferentiated hPSC detection, the detection sensitivities are partly constrained by the upper limits of the available experimental scale or by limits of the input sample amount. For example, the sensitivity of a culture assay for undifferentiated cell detection could be improved but might adversely impact the expense, time, and throughputs if cells are seeded into more plates, undifferentiated cells are concentrated through cell sorting, or differentiated cells are eliminated with apoptosis-inducible systems [20, 21]. Although these approaches imply that the sensitivity of the in vitro nucleic acid test could be improved by increasing the input amount of the sample, a conventional PCR is not ideal for testing a large amount of nucleic acids in a single reaction. For general quantitative PCR (qPCR), excessive input of nucleic acids is not recommended because too much nucleic acids causes nonspecific DNA amplification and/or a decrease in PCR efficiency that is far below the ideal two-folds per cycle. In the case of emerging digital PCR platforms, which are robust to interfering substances [22], the analytical performance is exacerbated as the input sample increases, and, in addition, highly depends on the desirable quantitative range and the occurrence of false-positive droplets, which lead to laborious validation assays and threshold selection [23–25].

Thus, for more precise, accurate, and sensitive detection of undifferentiated cells, we explored another nucleic acid amplification method capable of detecting subtle target RNAs among a vast amount of interfering substances and then determined that loop-mediated isothermal amplification (LAMP) [26] is the optimal solution. LAMP is known to be robust against interfering material due to the use of thermostable DNA polymerase from *Bacillus stearothermophilus* (*Bst*), which possesses strong DNA strand displacement activity. Furthermore, four primers that recognize six regions on a target nucleic acid insure highly specific target amplifications. Besides, the simple addition of thermostable reverse transcriptase enables rapid one-pot RNA detection by reverse transcription LAMP (RT-LAMP). Although LAMP is specialized to qualitative assays, the simple and rapid DNA amplification procedure enables a wide range of applications, including digital assays [27], lateral flow assays [28], and in situ target amplification. LAMP is especially suitable for the diagnosis of infectious disease as this technique has been adapted for the pulmonary tuberculosis detection kit recommended by World Health Organization (WHO) [29].

In this study, we aimed to establish a more sensitive and robust nucleic acid test platform for the detection of undifferentiated cells by increasing the input amount of the sample. Through evaluations using various combinations of pluripotency marker RNAs and differentiated cells, the adaptability of RT-LAMP for the tumorigenicity-associated test was investigated.

## Materials and Methods

### Cell Culture of hiPSC and hiPSC-derived HE, MH, EC, MC, and NPC

Ff-I01s04, Ff-I01, 1383D6, and 1231A3 hiPSCs were kindly provided by Center for iPS Cell Research and Application (CiRA) with the confirmed informed consent by the donors [30]. TkDA3-4 hiPSC was kindly provided by University of Tokyo [31]. HiPS-RIKEN-2A, HiPS-RIKEN-12A, and Nips-B2 hiPSCs were kindly provided by RIKEN Bio-Resource Research Center with the confirmed informed consent by the donors [32, 33]. The characteristics of the hiPSCs used were summarized in our previous investigation [34]. Mainly Ff-I01s04 was used for hiPSC spiked-in and differentiation. The over-passed Ff-I01 [17] was only used for preparing hepatic endodermal cells (HE) containing residual undifferentiated cells. The other hiPSC strains were used for the comparison of the pluripotency marker expressions in undifferentiated state. The hiPSCs were maintained on Laminin 511 E8-fragment-coated (iMatrix-511, kindly provided by Nippi) dishes with StemFit AK02N medium (Ajinomoto). hiPSC-derived definitive endodermal cells (DE), HE, immature hepatocytes (IH), mature hepatocytes (MH), mesenchymal cells (MC), and endothelial cells (EC) were obtained using cultivation protocols reported previously [35]. hiPSC-derived neural progenitor cells (NPC) were obtained from American Type Culture Collection (ATCC) and cultured following the ATCC manuals. The use of hiPSCs was approved by the ethical committee at Yokohama City University and the University of Tokyo.

### Cell Culture of Immortalized Cell Lines

293 T was kindly provided by RIKEN BioResource Research Center (RIKEN BRC) and HeLa by The European Collection of Authenticated Cell Cultures (ECACC). 293 T was plated on a 10 cm dish without coating and HeLa on one coated with 10 mL of 0.1% (w/v) porcine gelatin (Sigma-Aldrich) in PBS overnight at 37 °C. The cells were harvested in D-MEM high glucose (Wako) supplemented with 10% fetal bovine serum (FBS) (Funakoshi) and 1% (v/v) Pen Strep (Gibco). The cells were detached by 0.05% Trypsin–EDTA (Gibco).

### Undifferentiated hiPSC Spike-in and RNA Preparation for RT-LAMP Assay

The numbers of undifferentiated hiPSCs and differentiated cells were determined by a cell count using trypan blue staining. Next, 1.2 × 10^7^ differentiated cells were dispensed into each 15 mL conical tube and serial diluted (e.g., 10^0.5^-fold) undifferentiated hiPSC were spiked into differentiated cells. After centrifugation, the supernatant was disposed, and total RNA was prepared from a cell pellet using a PureLink RNA Mini Kit (Thermo Fisher Scientific). RNA was eluted with distilled water, and subsequently, RNA concentration was determined using NANODROP 2000c (Thermo Fisher Scientific) based on A_260_/A_230_ ratio.

### RT-qPCR

cDNA was obtained by reverse transcribing total RNA with a Thermal Cycler TP600 (Takara) and a High-Capacity cDNA Reverse Transcription Kit using random primers (Thermo Fisher Scientific). The obtained cDNA was used as a qPCR template and mixed with THUNDERBIRD Probe qPCR Mix (Toyobo), Taqman probe (Universal Probe Library; Roche), and gene-specific primers (Supplementary Table 1) following the manufacturers’ instructions. Eukaryotic 18S rRNA Endogenous Control (Thermo Fisher Scientific) was used as an internal reference, and thermal cycle reaction was performed using Light Cycler 480 systems (Roche). Per test, 12.5–30 ng of cDNA was assayed because a further increase of input led to early amplification of 18S. The reaction volume was 10 μL per test, unless otherwise stated, and the gene expression levels were quantified by ∆∆Cp method.

### LAMP Primer Design

LAMP primers of target genes were designed using Primer Explorer V5 (http://primerexplorer.jp/). LAMP primers and detection probes described in Supplementary Table 2 were synthesized by Eurofins Genomics and Japan Bio Services Co., Ltd. The primers successfully detected (i.e., to judge as positive) 10–50 copies of target per test in conditions that were free of interfering nucleic acids and did not produce any human genome DNA-derived amplicons. Furthermore, we added loop primers to accelerate and sensitize LAMP amplification [36].

### In vitro RT-LAMP

**Fig. 1:**
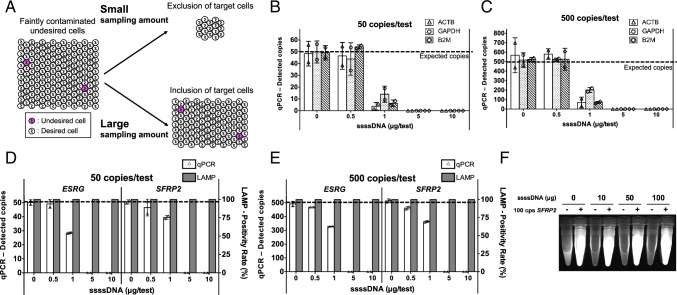
Sensitive and specific target detection facilitated by the robustness of LAMP against interfering nucleic acids. **A** Scheme depicting advantage of increasing input sample for detection of a few target cells among vast nontarget cells. **B** and **C** Exacerbation of qPCR quantitativity caused by interfering nucleic acids; 50 or 500 copies of artificial DNA of reference genes spiked into the indicated amount of ssssDNA were assayed by qPCR (*N* = 2 replicate measurements, mean ± SD). Dashed line shows expected copies. Primer sets were referenced from [37]. The reaction volume was 25 μL. **D** and **E** Specific detection of target nucleic acids by LAMP among interfering nucleic acids; 50 or 500 copies of target artificial DNA spiked into the indicated amount of ssssDNA were assayed by qPCR and LAMP (*N* = 2 replicate measurements, mean ± SD for qPCR). Dashed line shows expected copies for qPCR quantitation. The reaction volume was 25 μL for both qPCR and LAMP. **F** Sample containing 0 or 100 copies of *SFRP2* artificial DNA with 0, 10, 50, or 100 μg of ssssDNA were tested by LAMP. The labelled BLP was used and the reaction volume was 500 μL (*N* = 2 inter-day replicates, representative results were shown)

An in vitro RT-LAMP reaction buffer and RNA samples diluted with distilled water were prepared and mixed on ice in a Light Cycler® 480 Multiwell Plate 96 (Roche) or 8-Tube Strips (Roche). After vortex and centrifugation, one-step RT-LAMP was performed using a Light Cycler® 480 system with 25 μL reaction volume per well. Reverse transcription was performed at 55 °C for 10 min and, subsequently, an isothermal amplification was performed for 70 min. Fluorescence was measured through the amplification phase, and the threshold time values (Tt values) were determined by the time at which the baseline subtracted fluorescence intensity reached to − 1, except that of the multiplexed *LINC00678* reached to − 0.5, which reflected the probe annealing and quenching of fluorescent dye. For the multiplexed assay, Tt values were also determined based on the fluorescence increase of SYTO 63 intercalator by setting the threshold at 0.5. Tt values were set at 0 when the fluorescence did not reach the threshold intensity during reaction; instead, those were set at 80 (= 10 + 70) for nonparametric statistical analysis to rank data appropriately. The detailed buffer and reaction condition of LAMP is summarized in Supplementary Tables 3 and 4. For samples containing gene-specific artificial double-strand DNA templates containing the RefSeq RNA sequence shown in Supplementary Table 2, the samples were thermally denatured through incubation at 95 °C for 5 min and subsequently at 4 °C using TP600 prior to sample input. In some assays, salmon sperm single-strand DNA (ssssDNA) (Sigma) was used as a model for interfering nucleic acids. Only for Fig. 1F, LAMP reactions were performed with ALB-121 heat block (SCINICS) at 65 °C for 100 min. and images were obtained by WSE-6200 (ATTO).

### Culture Assay and Immunofluorescence Staining for Detection of Undifferentiated Cells

Undifferentiated cells were detected among over-passed hiPSC-derived HE by using a previously reported culture assay [16]. The collected differentiated cells were seeded on lamin511-E8-coated wells at a density of 8 × 10^4^ cells/cm^2^ with StemFit AK02N medium supplemented with Rock inhibitor Y-27632 (Wako) in 24-well plates. Thereafter, the AK02N medium was changed every day and cultured for 7–10 days to obtain undifferentiated cell colonies of an appropriate size. After cultivation, the cells were treated first with 4% (v/v) paraformaldehyde (Wako) for 15 min and then with 0.5% (v/v) Triton-X100 (Sigma-Aldrich) in phosphate-buffered saline solution (PBST) for 10 min. After blocking with PBST supplemented with 5% (v/v) FBS (blocking buffer), the cells were immersed in a blocking buffer with primary anti OCT4A or anti SOX2 antibodies at 4 °C overnight and then with fluorolabelled secondary antibodies for 1 h. The antibodies and their dilution rates are summarized in Supplementary Table 5. Nuclei were stained with 4’,6-diamidino-2-phenylindole (DAPI) (Dojindo) in Apathy’s mounting media (Wako). The fluorescent images were obtained with fluorescence microscopes BZ-X710 (Keyence) and cellSens (Olympus).

### In situ RT-LAMP

HE, MC, or EC containing appropriate numbers of spiked-in hiPSCs were cultured, fixed, and permeabilized as prepared for the above culture assay in a 96-well plate. Then, 50 μL of RT-LAMP reaction mix containing fluorolabelled loop primers or alkali-stable Digoxigenin (DIG)-11-dUTP (Sigma-Aldrich) was added, and the well was sealed with Light Cycler® 480 sealing foil (Roche). The plate was incubated at 56 °C for 30 min and 63 °C for 90 min on a slide warmer PS-53 (Sakura Finetek). Subsequently, the wells were washed with PBS, and DIG or pluripotency marker proteins were immunostained following the aforementioned method (Supplementary Table 5) prior to image acquisition. Detailed buffer conditions of LAMP are shown in Supplementary Table 6.

## Results

### Increased Amount of Input Sample for Sensitive Target Cell Detection

To detect a subtle target molecule by distinguishing it from a vast amount of nontargets, the input amount of sample should be increased so as not to lose targets. Due to the cell-based environment (Fig. 1A), we hypothesized that the sensitivity and reproducibility of nucleic acid tests could be improved by maximizing the input amount of sample so long as the test results are not influenced by the interferents. However, due to the obvious exacerbations of qPCR quantitativity in conditions with more than 1 µg interfering nucleic acids per test (Figs. 1B and 1C), we constructed RT-LAMP assays (Supplementary Fig. 1 shows the principle) anticipating sensitive and specific target amplification from a vast number of interfering materials. Indeed, the comparison of qPCR and LAMP clearly showed the superiority of LAMP in detecting 50 and 500 copies of targets even in the presence of 10 µg interfering nontarget nucleic acids in a mere 25 µL reaction volume (Figs. 1D and 1E).

Furthermore, toward sensitive undifferentiated cell detection among differentiated cells, if no *SFRP2* RNA were expressed in differentiated cells, hypothetically, more than 50 copies of *SFRP2* RNA from a single hiPSC contamination [17] would be enough for LAMP to obtain positive results by inputting all the extracted total RNA to a single reaction. Generally, the mass of total RNA in a human cell is known to be 10–30 pg [38], although this estimation depends on the cell type [39]. Thus, we assumed the amount to be 10 pg total RNA per cell, and 100 copies of the target were spiked into 100 μg interfering nucleic acids, modeling two hiPSCs contamination per 1 × 10^7^ differentiated cells. This sample was assessed by LAMP and resulted in positive reaction, which indicated the LAMP could detect 0.00002% hiPSC contamination (Fig. 1F). This robustness against interfering materials to achieve sensitive and specific target detection and the scalability of the isothermal reaction suggested a novel LAMP application for tumorigenicity-associated assays of cell-derived materials.

### Pluripotency Marker RNAs Suitable for RT-LAMP

For sensitive, robust tumorigenicity-associated assays, pluripotency markers should be highly and specifically expressed in undifferentiated hPSCs but hardly expressed in desired differentiated cells (Supplementary Fig. 2). In hiPSC-derived HE, MC, and EC, *OCT4*, *SOX2*, *NANOG*, and *LIN28A*, the representative pluripotency RNAs, were not applicable for sensitive detection of residual undifferentiated cells because the expressions of these RNAs remain high in these cells, which hinders sensitive hPSC detection. However, *ESRG* and *SFRP2*, which are the pluripotency marker genes that are useful for qPCR-based assays, had lower expressions in these differentiated cells [17] (Supplementary Fig. 2C). These findings reminded us of the utility of pluripotency marker genes, which have little or no expression in desired differentiated cells.

Accordingly, after analyzing the single-cell transcriptome data of hiPSC and HE through read re-mapping and sashimi plotting [40], expression of 45 candidate genes shown in Supplementary Fig. 3 were assessed by RT-qPCR using RNA extracted from hiPSC, DE, HE, IH, MH, or HE spiked with hiPSC. This screening revealed the utility of *PR/SET domain 14* (*PRDM14*) and *long intergenic non-protein coding RNA 678* (*LINC00678*) for RT-qPCR assay (Supplementary Fig. 4). Specifically, the expression of *LINC00678* was subtle in all assessed differentiated cells, which indicates its potential as another reliable marker (Supplementary Figs. 2C and 2D). In addition, these genes were commonly and highly expressed in the seven hiPSC strains, which suggested wide adaptability of the markers (Supplementary Fig. 5).

Subsequently, we simulated hiPSC detection sensitivity to estimate the advantage of increasing the input amount of RNA per test by RT-LAMP in comparison with RT-qPCR. Pluripotency markers with extremely high expression in hiPSC, like *SFRP2* and *LIN28A*, would be more than 50 copies per hiPSC, whereas other pluripotency markers of interest would be more than 5 copies per hiPSC. Regarding those expressions in differentiated cells, markers that are expressed extremely low, like *ESRG* and *LINC00678*, could be less than 1 copy per 10^5^ differentiated cells, whereas other pluripotency markers could be less than 1 copy per 10^4^ differentiated cells.

Based on these parameters, RT-qPCR performed with 50 ng total RNA per test would enable 0% to be distinguished from 0.01% hiPSC contamination, although it would be hard to discriminate 0% from 0.001% contamination, thereby resulting in a detection limit within the range as reported by articles on *ESRG* and *LIN28A* [17–19], based on the estimated qPCR detection limit, which was three copies per test (Supplementary Fig. 6) [41]. With RT-LAMP, we can control positive/negative cutoff copy number of target RNA per test between approximately 10 and 1,000 copies per test by adjusting the reaction buffer (Supplementary Fig. 7) and temperature conditions [42]. This adjustability of the qualitative LAMP cutoff would enable 0% hiPSC contamination to be distinguished as negative and 0.001% contamination as positive based on several marker genes if the RT-LAMP assays were performed with 5 µg total RNA per test (Supplementary Fig. 6).

### Detection of Residual Undifferentiated Cells Remaining after hiPSC Differentiation

Next, we assessed whether the RT-LAMP assay discriminates HE free of undifferentiated cells from HE containing residual undifferentiated cells by simulating a practical hiPSC differentiation process. In our routine HE, MC, and EC differentiation processes, no residual undifferentiated cells were observed when culturing the commonly used hiPSC strains [35]. Thus, we employed the over-passed hiPSC as a model strain, which tends to induce the remaining undifferentiated cells in our HE differentiation process [35] and compared the results of the culture assay, RT-qPCR, and RT-LAMP targeting three pluripotency marker genes, *ESRG*, *LINC00678*, and *PRDM14*.

**Fig. 2:**
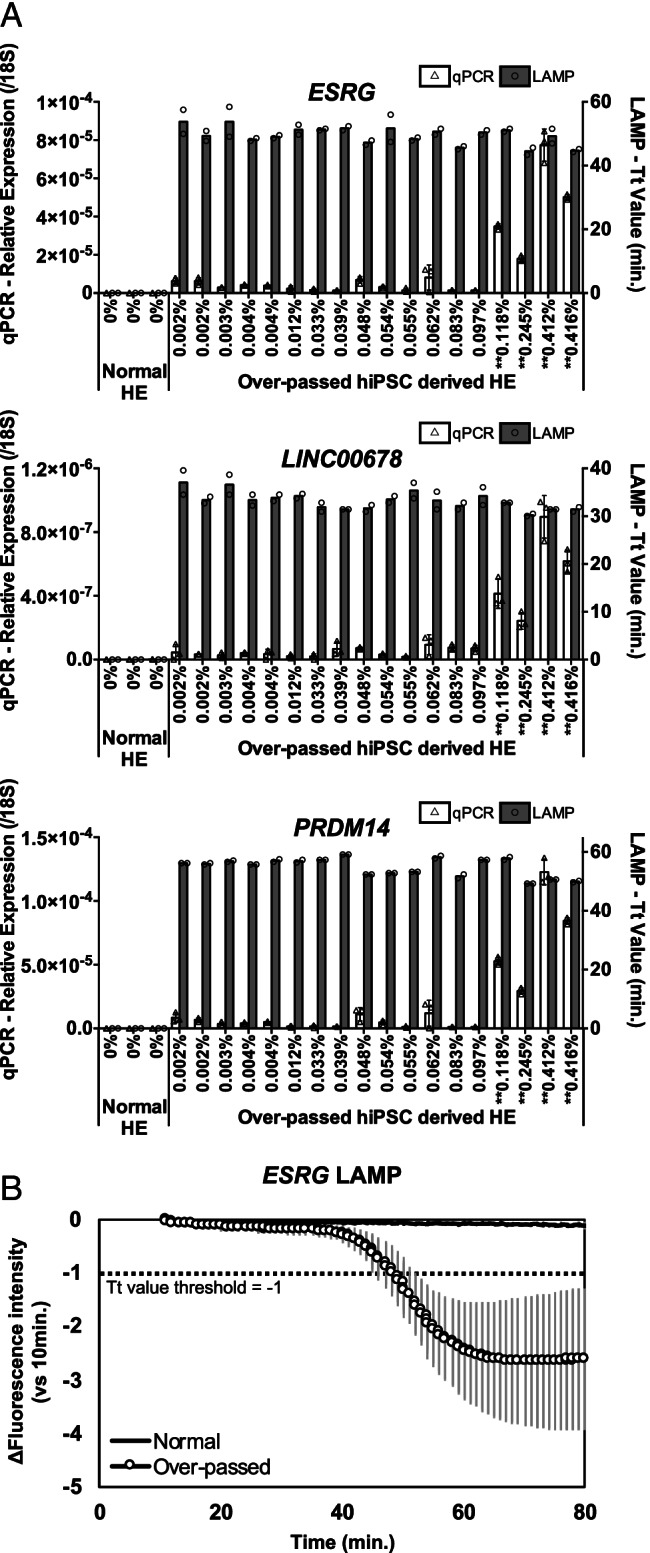
Discrimination between HE with and without residual undifferentiated cells by RT-LAMP. **A** Normal hiPSC and over-passed hiPSC were differentiated into HE and tested by culture assay, RT-qPCR, and RT-LAMP. The residual undifferentiated cells per HE ratio determined by culture assay are shown below the bars. RT-qPCR or RT-LAMP was performed with 12.5 ng or 1 µg total RNA per test in this experiment, respectively. (*N* = 2, 3, or 2 replicate measurements for culture assay, RT-qPCR, or RT-LAMP, respectively; mean ± SD values for RT-qPCR; median values for RT-LAMP; Tukey–Kramer test vs. Normal HE for RT-qPCR, *: *p* < 0.05, **: *p* < 0.01 shown beside the residual undifferentiated cells per HE ratio). **B** Representatively, the time course of *ESRG* RT-LAMP is shown (*N* = 6 or 36 for normal or over-passed hiPSC-derived HE corresponding to each data plot in the bar graph in (A); mean ± SD for each time point)

As expected, although HE derived from normal hiPSC did not contain any undifferentiated cells, those derived from over-passed hiPSC led to undifferentiated cell colony formations. Based on the observed colony number, the residual rates of undifferentiated cells were calculated (Supplementary Table 7), although the culture assay may have some quantitative limitations because not all the seeded undifferentiated cells form colonies, especially when the number of residual undifferentiated cells was small [43]. In this condition, RT-qPCR did not distinguish some residual undifferentiated cells, particularly if they comprised below 0.1% of the seeded cells, likely due to low target RNA copy numbers per test and measurement error in this low copy number range (Fig. 2). However, RT-LAMP assays, whose cutoffs were set between 100 and 1,000 copies per test (Supplementary Fig. 7), clearly discriminated between samples containing no undifferentiated cells and samples containing 0.001% or more undifferentiated cells, benefiting from the 80-fold greater RNA input per test than RT-qPCR.

### Sensitive Detection of Spiked-in hiPSCs Among Differentiated Cells by RT-LAMP

**Fig. 3:**
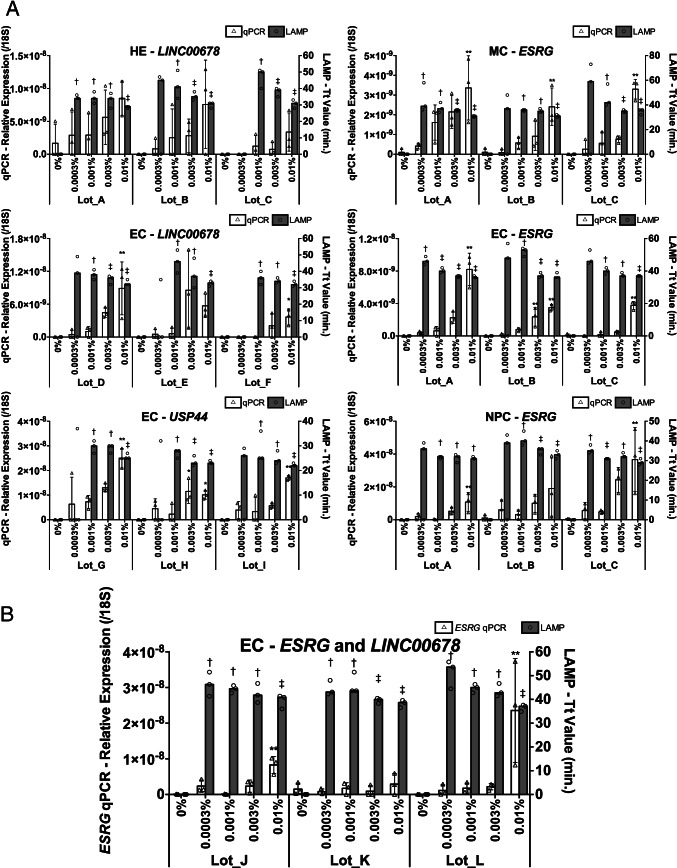
Sensitive detection of spiked hiPSC among hiPSC-derived HE, MC, EC, or NPC by RT-LAMP. hiPSC was spiked into hiPSC-derived cells and then extracted total RNA were assayed by RT-qPCR using 30 ng per test and RT-LAMP using 5 μg per test (*N* = 3 biological replicates × 3 replicate measurements, mean ± SD values for RT-qPCR and median values for RT-LAMP, Tukey–Kramer test vs. 0% of each cell lot for RT-qPCR, *: *p* < 0.05, **: *p* < 0.01, Shirley–Williams test vs. 0% of each cell lot for RT-LAMP, †: *p* < 0.05, ‡: *p* < 0.01). For RT-LAMP, each target gene was analyzed (**A**) by probe detection, whereas *ESRG* and *LINC00678* were multiplexed in (**B**), and the Tt values were determined based on the fluorescence of SYTO63 intercalator

To confirm the hiPSC detectability of our RT-LAMP systems, we compared the sensitivity between RT-qPCR and RT-LAMP assays by using total RNA extracted from differentiated cells containing spiked-in hiPSCs. Firstly, serially diluted hiPSCs were spiked into hiPSC-derived endodermal, mesodermal, and ectodermal cells. To optimize the RT-LAMP conditions, the reaction conditions were finely tuned for each target gene and cell so as to discriminate the hiPSC spiked-in samples from hiPSC-free samples. By inputting 5 μg total RNA per test, we determined the RT-LAMP conditions that could detect 0.001% hiPSC contamination (Fig. 3A). RT-qPCR using the same RNA samples further revealed the superior hiPSC detection sensitivity of RT-LAMP in concordance with our above simulation results (Supplementary Fig. 6).

Subsequently, because we anticipated that RT-LAMP is also applicable for the evaluation of a wide range of differentiated cells, 293 T and HeLa cells were additionally examined as model human differentiated cells, assuming that cross-contamination of cells had occurred [44, 45]. This investigation also indicated the high sensitivity of RT-LAMP and that proper pluripotency marker selection enables the evaluation of faint hiPSC contamination in various cell-derived materials in addition to hPSC-derived materials (Supplementary Fig. 8).

Finally, for more robust detection of undifferentiated cells, we developed a multiplex RT-LAMP assay targeting *ESRG* and *LINC00678*, keeping the high detection sensitivity without any nonspecific amplification nor nonspecific probe hybridization (Fig. 3B). In these multiplexed measurements, although we anticipated obtaining either *ESRG*( +)/*LINC00678*( −) or *ESRG*( −)/*LINC00678*( +) wells as positive wells as the two genes are amplified competitively in each well, some *ESRG*( +)/*LINC00678*( +) wells were also observed, especially in samples containing more spiked-in hiPSCs (Supplementary Fig. 9), which possibly reflected the balanced abundance of the two targets in extracted RNA (Supplementary Fig. 2B). Altogether, through assessing more than 1 μg of total RNA per test, RT-LAMP achieved a highly sensitive detection of undifferentiated cells among various differentiated cells, which indicated the applicability of RT-LAMP for tumorigenicity-associated assay.

### In situ RT-LAMP Labeling of Undifferentiated Cells and Co-Staining with Pluripotency Proteins

As a further application, the advantages of LAMP and the extremely low expressions of pluripotency marker RNAs in differentiated cells indicated that imaging-based detection of hPSCs was viable. The robustness against interference and high specificity of LAMP enabled in situ detection of nucleic acids originating from pathogens and physical disorders [46–48]. Compared with in situ PCR, a relatively mild isothermal reaction could be performed with simple apparatuses and presumably circumvents heat damage to the biological analytes. Thus, we supposed in situ RT-LAMP could detect undifferentiated cell colonies among hiPSC-derived differentiated cells in the culture assay process, as well as immunostaining of pluripotency protein markers.

**Fig. 4:**
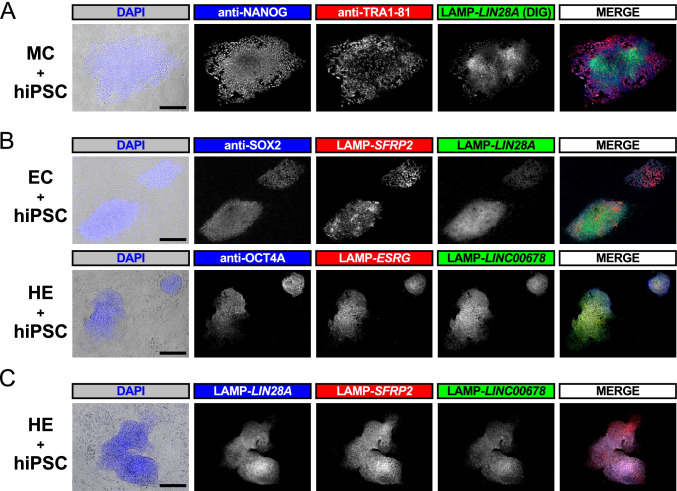
Specific detection of undifferentiated cells by in situ RT-LAMP. Undifferentiated cell colony detection by immunostaining and/or in situ RT-LAMP in the culture assay process visualizing DIG-labeled (**A**), double (**B**), or triple (**C**) fluorolabelled LAMP amplicons. In (A) and (B), pluripotency marker proteins were co-stained by immunostaining (Scale bar, 200 μm.). The whole well images are also shown in Supplementary Fig. 10. Compared to EC and colonies of hiPSC, HE and MC showed weaker DAPI signal presumably because these cells could not adapt to AK02 media during the incubation for undifferentiated cell colony formation

In the process of condition screening, we found that immunostaining of the five pluripotency marker proteins, OCT4A, SOX2, NANOG, SSEA4, and TRA1-81, produced substantial fluorescent signals after the 2-h heat treatment necessary for RT-LAMP. Thus, we co-stained the various combinations of pluripotency proteins and DIG- or fluorolabelled LAMP amplicons for the specific detection of undifferentiated cells (Fig. 4 and Supplementary Fig. 10). Intriguingly, some undifferentiated cell colonies were labeled nonuniformly in a speckled manner by in situ RT-LAMP (Fig. 4A and Supplementary Fig. 10C), which possibly reflected the focally exponential target amplification in the later amplification steps of LAMP (Supplementary Fig. 1). These specific undifferentiated cell labeling techniques further convinced the sensitivity and specificity of our LAMP constructs and the specific abundance of pluripotency marker RNAs in undifferentiated cells.

## Discussion

Among various hazardous impurities in cell-derived materials for regenerative medicine treatment, the detection of tumorigenic undifferentiated cells requires sensitivity improvements due to their risk and rare proportion in the desired material. We hypothesized that an increase of input sample per test would enhance the detection sensitivity of an in vitro assay and explored nucleic acid amplification strategies alternative to PCR. We focused on the unique characteristics of LAMP, developed RT-LAMP systems that target six pluripotency marker RNAs, and achieved sensitive detection of undifferentiated cells among hiPSC-derived endodermal, mesodermal, and ectodermal cells.

The hiPSC detection sensitivity of in vitro RT-LAMP assay is presumed to be at least 0.00002%, and higher sensitivity can be achieved by maximizing the input RNA amount. Using hiPSC-derived cells and model differentiated cell lines with several marker RNAs, the hiPSC detection sensitivity was 0.001%, which was more sensitive compared with that of RT-qPCR [17] and was comparable with the reported digital RT-PCR assays targeting *LIN28A* or *TDGF1* [18, 19], with the simple and rapid one-pot assay process. In addition, the sensitive multiplexed assay and specific in situ RT-LAMP labeling of undifferentiated cells further clarified the utility of pluripotency marker RNAs and RT-LAMP. Our attempts indicated a novel solution for simple and sensitive evaluations of hPSC contamination in cell-derived products and residual undifferentiated cells among hPSC-derived materials.

Our group is preparing for clinical trials involving a liver failure treatment using all hiPSC-derived liver buds (LBs). Based on the coculture technology of hiPSC-derived HE, MC, and EC [49], mass production of LB organoids through chemically defined animal origin-free differentiation has been achieved [35, 50]. As the transplanted LBs consisted of 10^8^–10^10^ cells to be engrafted on the recipients’ liver for years, the tumorigenicity risk has been cautiously assessed through functional evaluation of LBs and validation of operational procedures using mice, rat, and porcine models [51, 52]. Furthermore, based on the in vitro assays, the use of pluripotency markers and RT-LAMP presented in this study further ensures the safe application of our LBs.

We utilized RT-LAMP assay targeting six pluripotency RNA markers and assessed six types of differentiated cells, which indicates the wide adaptability of this technique for the evaluation of various cell-derived materials. Because the expression level of each pluripotency marker RNA would widely differ among cell therapy products, prior marker gene selection would be mandatory for any nucleic acid tests. In this study, the RNA detection sensitivity—alternatively, the qualitative cutoff copy number of RNA—of RT-LAMP was deliberately determined for each marker gene and differentiated cell to discriminate between 0.001% and no hiPSC contamination. Nevertheless, for more practical assay development toward regulatory science, the cutoff values could be set beforehand for each marker gene from the risk and hazard perspectives of each cell therapy product; for instance, at 50 copies per 5 μg total RNA or at 50 copies per 1 μg total RNA, which exceed the general measurement ranges of qPCR and digital PCR. In principle, although the LAMP approaches have the potential to achieve 0.00002% or a more sensitive detection limit of undifferentiated cells, the sensitivity improvement was mainly hindered by the slight expression of pluripotency marker RNA from the differentiated cells used in hiPSC spike-in assays. To repress these background expressions, further refinements to the differentiation protocol [34] or simple techniques for the elimination of intermediate and immature cells would be effective. Plus, additional LAMP buffer condition screening would enable increase of input amount of RNA to improve sensitivity and reproducibility.

Further technical improvement of RT-LAMP tumorigenicity-associated tests could involve a more rational selection of primer positions to optimize performance. Some pluripotency genes, such as *OCT4* and *NANOG*, are known to have many pseudogenes and/or their cognate sequences all over the genome and those expression of RNA variants could be altered in a differentiation-dependent manner [53, 54]. Among the pluripotency marker RNAs assessed by LAMP, *ESRG*, *LINC00678*, *PRDM14*, and *LIN28A* have such homologous sequences on the genome that those transcripts might affect the performance of nucleic acid tests. Further, cutting-edge single-cell transcriptome studies revealed not only transitions of RNA expression but also alterations of RNA structures, including splicing variants and splicing intermediates, along hPSC differentiation [55, 56]. These differences in RNA structure throughout the cell differentiation process could refine RNA-based assays; we located the PCR and LAMP primers for *USP44* at exon1-2 junction [57] and for *PRDM14* at exon2-3 junction [58], which are more abundant in hPSCs than in differentiated or matured cells based on RNA structural studies [59]. Additional accumulations of single-cell and long-read RNA sequence data will support the discovery of novel pluripotency marker genes and their appropriate primer positions.

Although RT-LAMP offers simple and sensitive in vitro tumorigenicity-associated tests, the requisition for a large amount of RNA might limit its use, especially for cell therapy products that require a small number of cells and are prepared at small scales. In contrast, this technique would be preferable for products that are manufactured in large batches, such as allogenic cell products, or when large numbers of cells are used to treat patients. Altogether, qPCR, digital PCR, LAMP, and other nucleic acid amplification methods possess different pros and cons [60], and their usage should be determined comprehensively.

Because regulatory science in regenerative medicine field requires handling various cell therapy products and creating innovative evaluation technologies, further interdisciplinary collaboration would lead to remarkable solutions. In particular, an increase in the input amount samples or an efficient scale up of assays would be a pivotal approach for the sensitive measurement of subtle target analytes. Although LAMP assays have been used globally in the clinical diagnosis of infectious diseases, genotyping, food safety, and environmental assessment [61–67], this study is the first to apply the LAMP approach to regenerative medicine, which paves the way for novel applications of isothermal nucleic acid amplification methods. The use of LAMP assays is not limited to undifferentiated cell detection but would be applicable for various evaluations of a wide range of cell materials used in industries like Chinese hamster ovary, baby hamster kidney, or Vero cells, and those treated for human like chimeric antigen receptor T (CAR-T) or blood cells through sensitive and specific target detection.

## Supplementary Information

Below is the link to the electronic supplementary material.

Supplementary file1 (PDF 3.12 mb)

## Data Availability

The data generated during and/or analyzed during the current study are available from the corresponding author on reasonable request.
