# Supplementary material for: Highly Sensitive Detection of Human Pluripotent Stem Cells by Loop-Mediated Isothermal Amplification
Source: Stem Cell Rev Rep. 2022 Jun 3;18(8):2995–3007. doi: 10.1007/s12015-022-10402-3 (PMC9622575; doi:10.1007/s12015-022-10402-3)
Supplement: Supplementary file 1 — Supplementary file1 (PDF 3.12 mb) [file 12015_2022_10402_MOESM1_ESM.pdf]

Supplementary Materials

Highly sensitive detection of human pluripotent stem cells by loop-mediated isothermal amplification

Ryota Yasui, Atsuka Matsui, Keisuke Sekine, Satoshi Okamoto, Hideki Taniguchi

Corresponding authors

Keisuke Sekine

E-mail: kesekine@ncc.go.jp; ksekine@yokohama-cu.ac.jp

Hideki Taniguchi

E-mail: rtanigu@ims.u-tokyo.ac.jp; rtanigu@yokohama-cu.ac.jp

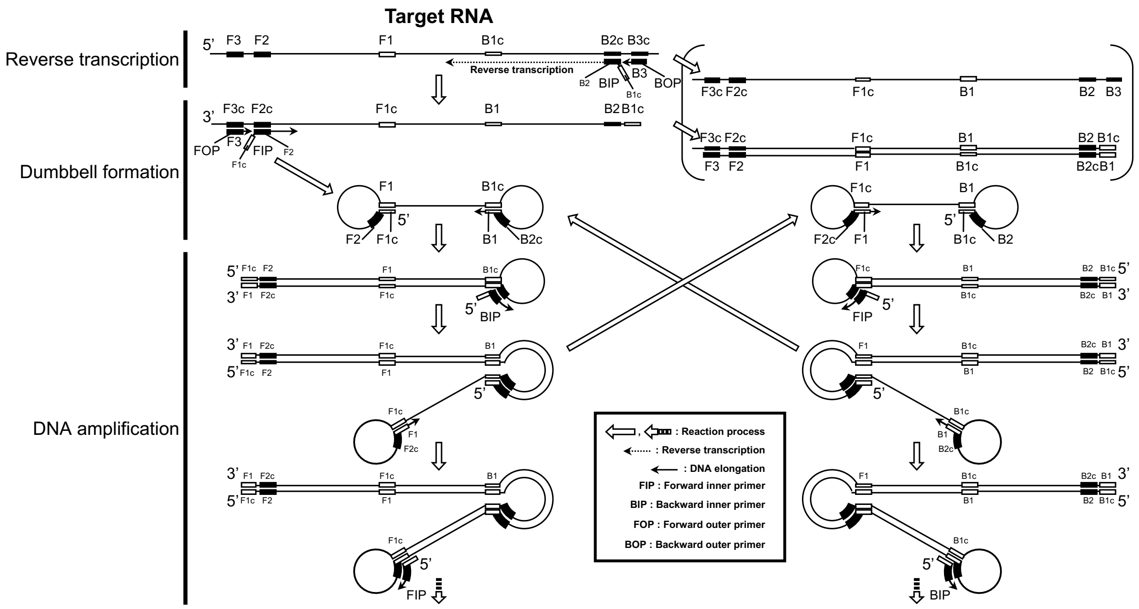

Supplementary Figure 1. Principle of RT-LAMP.

White arrows show reaction process. Black dashed arrow shows cDNA synthesis. Black arrows show DNA elongation facilitated by the strand displacement activity of *Bst* DNA polymerase depending on four primers: forward inner primer (FIP), backward inner primer (BIP), forward outer primer (FOP), and backward outer primer (BOP). Biproducts are shown in black brackets. Target RNA could be both plus- and minus-strand likewise, although this scheme only starts from plus-strand RNA. Template RNA is to be degraded due to RNase H activity of reverse transcriptase.

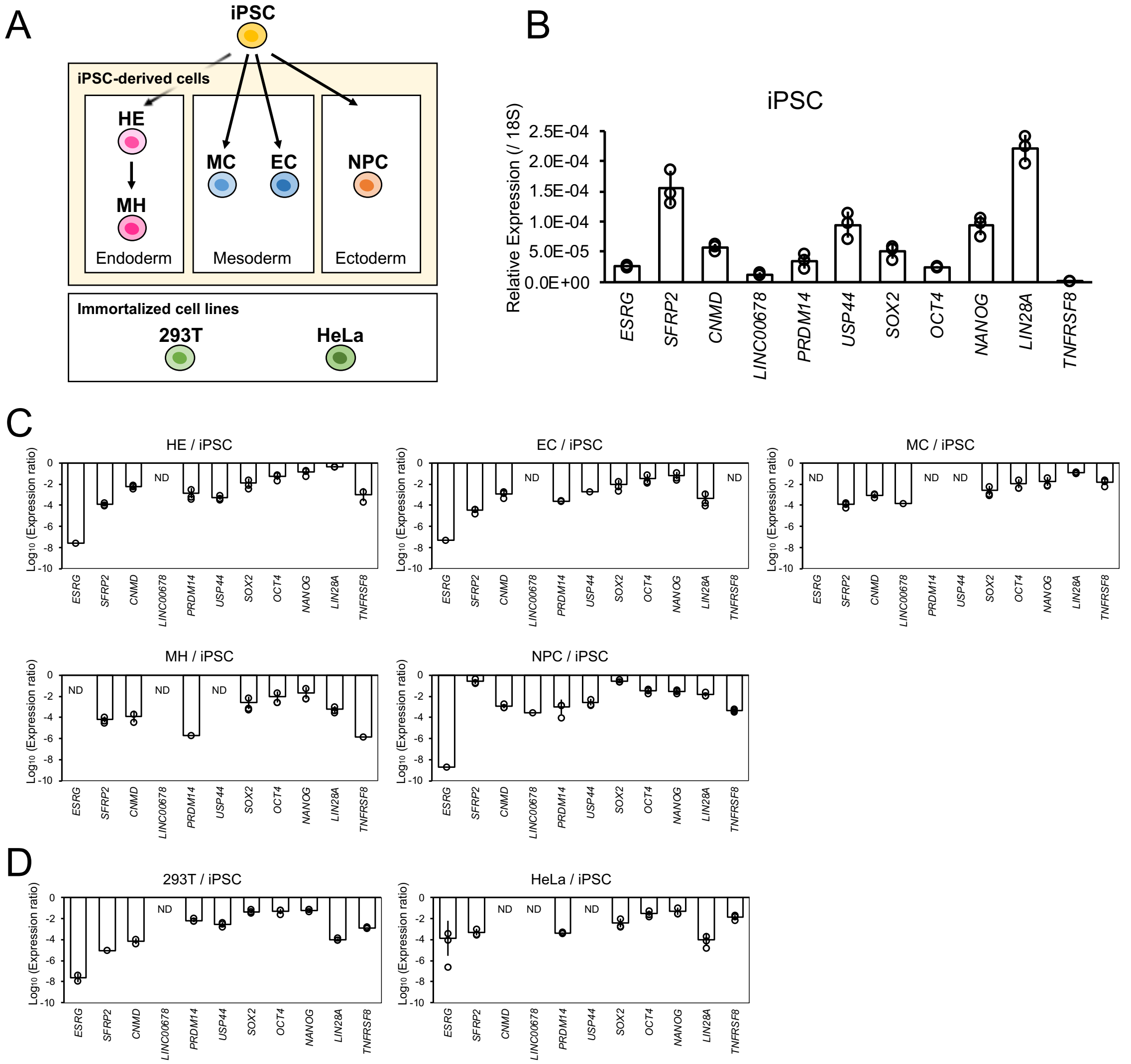

Supplementary Figure 2. Cells and pluripotency marker genes used in this study.

(A) Cells used in this study. (B) Expression levels of pluripotency marker RNAs in hiPSC determined by RT-qPCR (*N* = 3 biological replicates, mean ± SD). (C and D) Relative expression levels of pluripotency marker RNAs in hiPSC-derived differentiated cells (C) and immortalized cell lines (D) compared with that of hiPSC determined by RT-qPCR (*N* = 3 biological replicates, mean ± SD, ND: not detected).

A

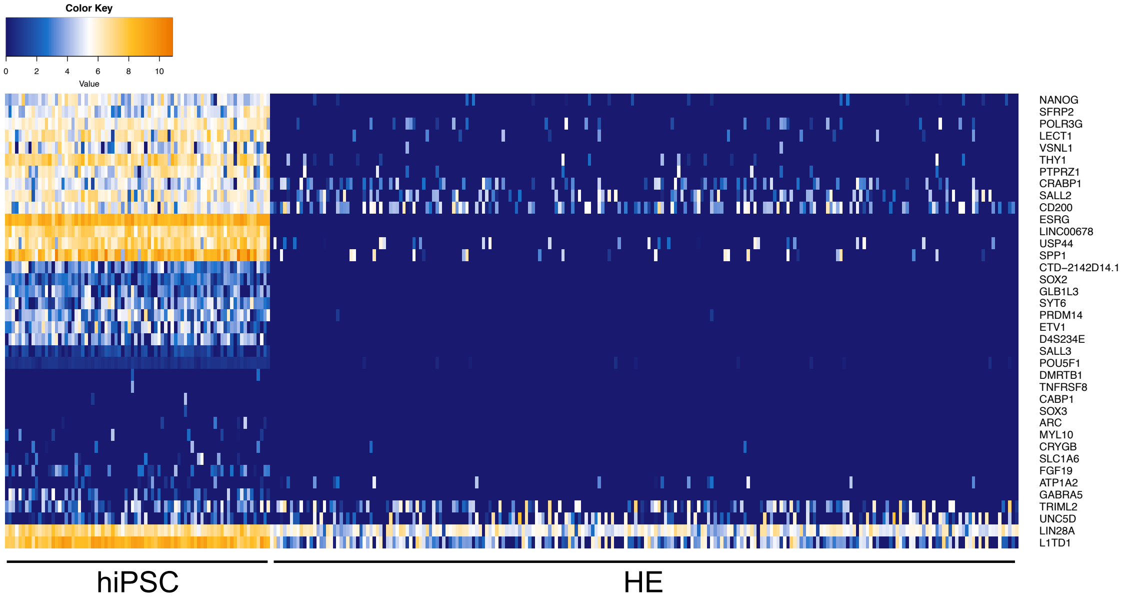

B

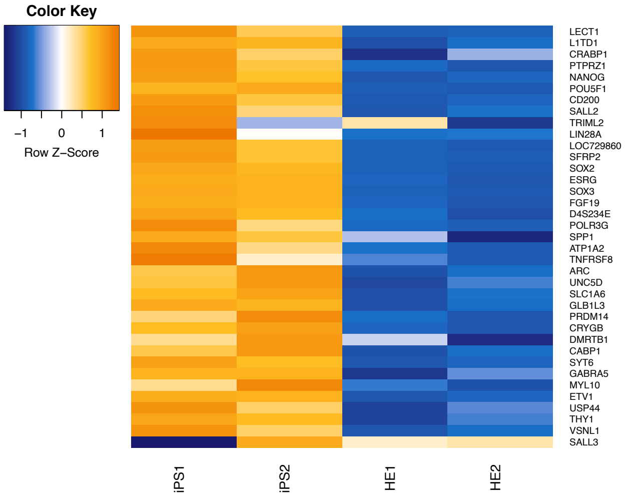

Supplementary Figure 3. Pluripotency marker gene candidates screened in this study. Genes expressed highly in hiPSC and lowly in HE were selected and their expression levels were shown as heatmaps based on (A) microarray or (B) single-cell RNA sequencing data. The detail of the transcriptome analysis was reported previously [17,39]. Some genes whose expression levels could not be analyzed were excluded from either heatmaps.

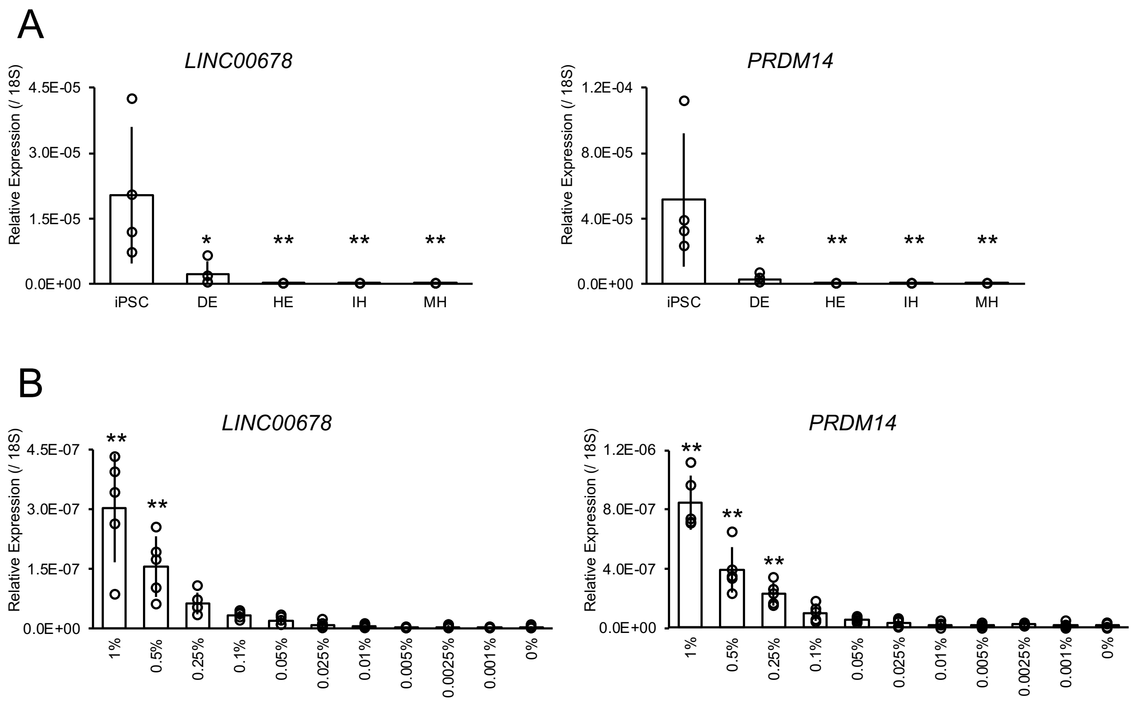

Supplementary Figure 4. Pluripotency marker RNAs, *LINC00678* and *PRDM14*, for sensitive detection of hiPSC among HE.

(A) Expression levels of *LINC00678* and *PRDM14* in hiPSC-derived hepatic lineage cells determined by RT-qPCR. hiPSC was differentiated into DE, HE, IH and MH via 6-, 10-, 13-, 21-day cultivation, respectively. (N = 4 biological replicates, mean ± SD, Tukey–Kramer test vs. hiPSC, * : $p < 0.05$, ** : $p < 0.01$). (B) hiPSC detection sensitivity of RT-qPCR targeting *LINC00678* and *PRDM14.* hiPSC was spiked into HE and extracted RNA were assayed (N = 5 biological replicates, mean ± SD, Tukey–Kramer test vs. 0%, * : $p < 0.05$, ** : $p < 0.01$).

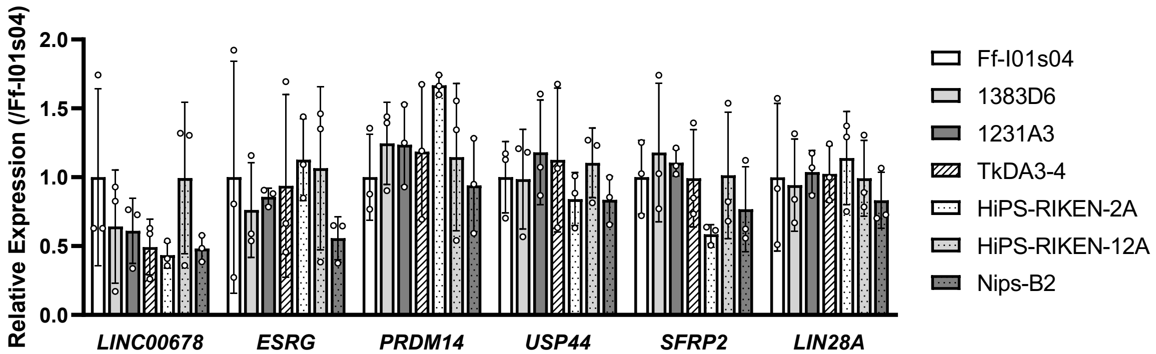

Supplementary Figure 5. Expression of pluripotency marker RNAs for RT-LAMP in various hiPSC strains.

Expression levels of *LINC00678, ESRG*, *PRDM14, USP44, SFRP2,* and *LIN28A* in seven undifferentiated hiPSC strains determined by RT-qPCR (*N* = 3 biological replicates, mean ± SD). No significant difference was observed between any of the hiPSC strains by Kruskal-Wallis test.

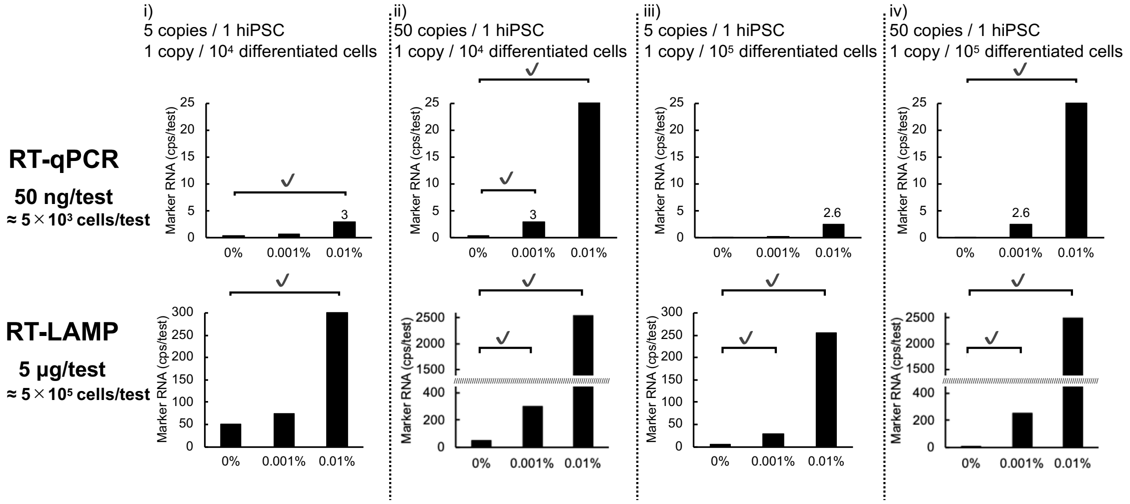

Supplementary Figure 6. Estimations of hiPSC detectability of RT-LAMP.

Improvement of hiPSC detection sensitivity by increasing the RNA input amount. The estimated copy number of RNA per test was simulated based on the number of differentiated cells, hiPSCs per differentiated cell ratio, and copies number of pluripotency marker RNA in hiPSC and in differentiated cell; the four postulated patterns of pluripotency marker expressions are shown as i)–iv) above the graphs.

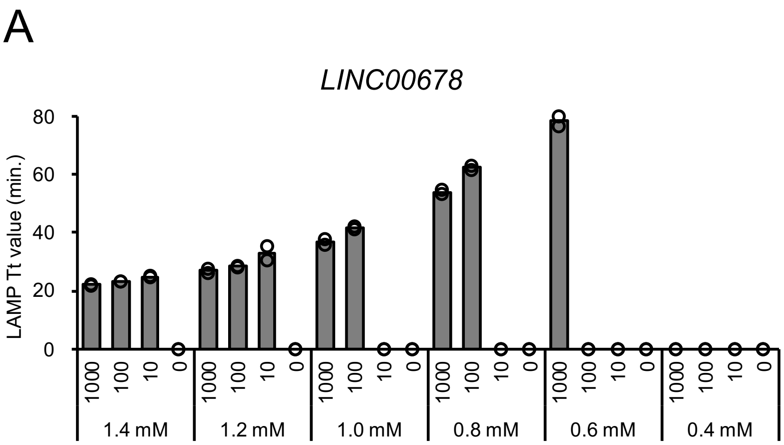

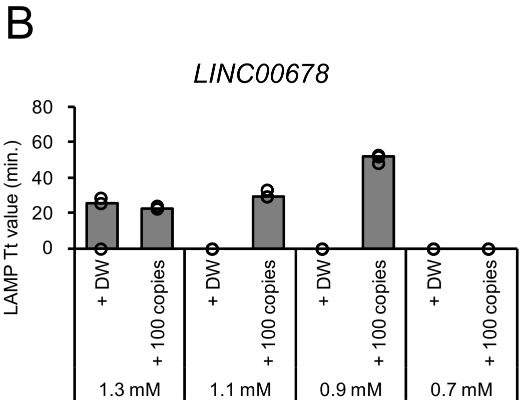

Supplementary Figure 7. Sensitivity adjustment of RT-LAMP by dNTPs.

(A) Discrimination between 1000, 100, 10, and 0 copies of artificial DNA (*N* = 2 replicate measurements, median values). (B) Discrimination between 1 μg of total RNA extracted from HE with or without 100 spiked-in copies of *LINC00678* artificial DNA (*N* = 3 replicate measurements, median values). Although 1.1 and 0.9 mM dNTPs led to the discrimination of the artificial DNA addition, 1.3 and 0.7 mM dNTPs failed.

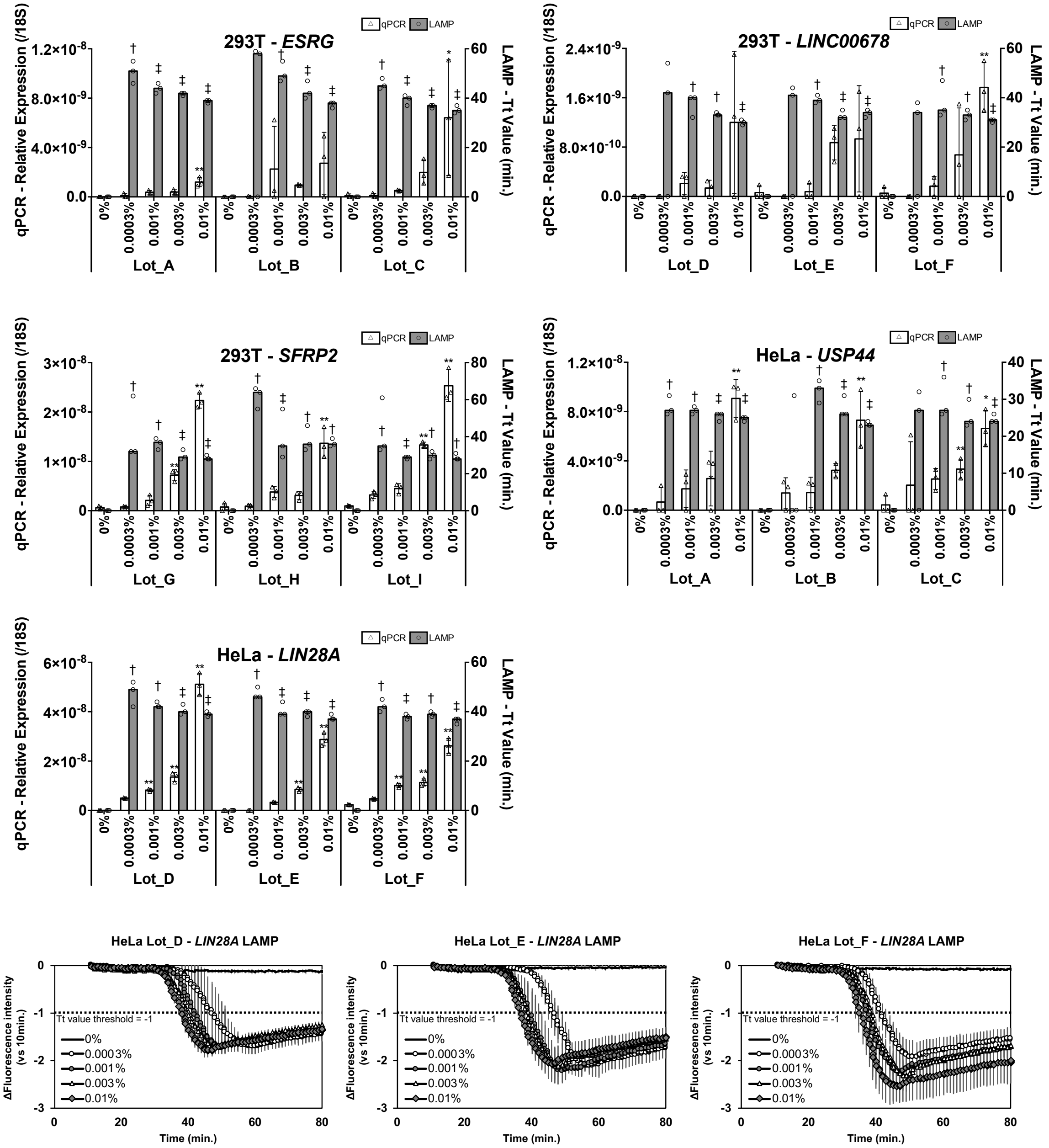

Supplementary Figure 8. Sensitive detection of spiked-in hiPSCs among model immortalized cell lines by RT-LAMP.

hiPSC was spiked into 293T or HeLa and then extracted RNA were assayed by RT-qPCR and RT-LAMP (N = 3 biological replicates × 3 replicate measurements, mean ± SD for RT-qPCR and median values for RT-LAMP, Tukey–Kramer test vs. 0% of each cell lot for RT-qPCR, * : $p < 0.05$, ** : $p < 0.01$, Shirley–Williams test vs. 0% of each cell lot for RT-LAMP, † : $p < 0.05$, ‡ : $p < 0.01$). Representatively, the time course of the LAMP reaction assessing *LIN28A* using HeLa RNA were also shown (N = 3 replicate measurements, mean ± SD for each time point).

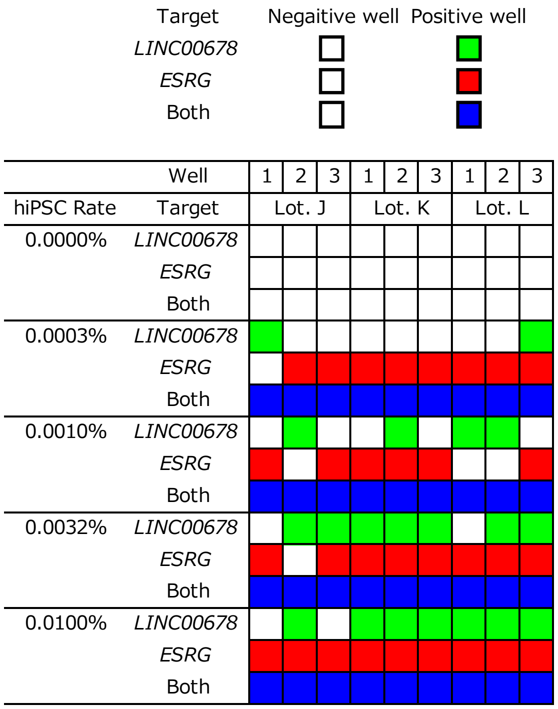

Supplementary Figure 9. Sensitive detection of spiked hiPSC among hiPSC-derived EC by multiplexed RT-LAMP.

Qualitative results of the respective well and the detection channel. Amplification of *LINC00678*, *ESRG*, or either of these two genes were monitored using 510, 580, or 660 nm detection filter.

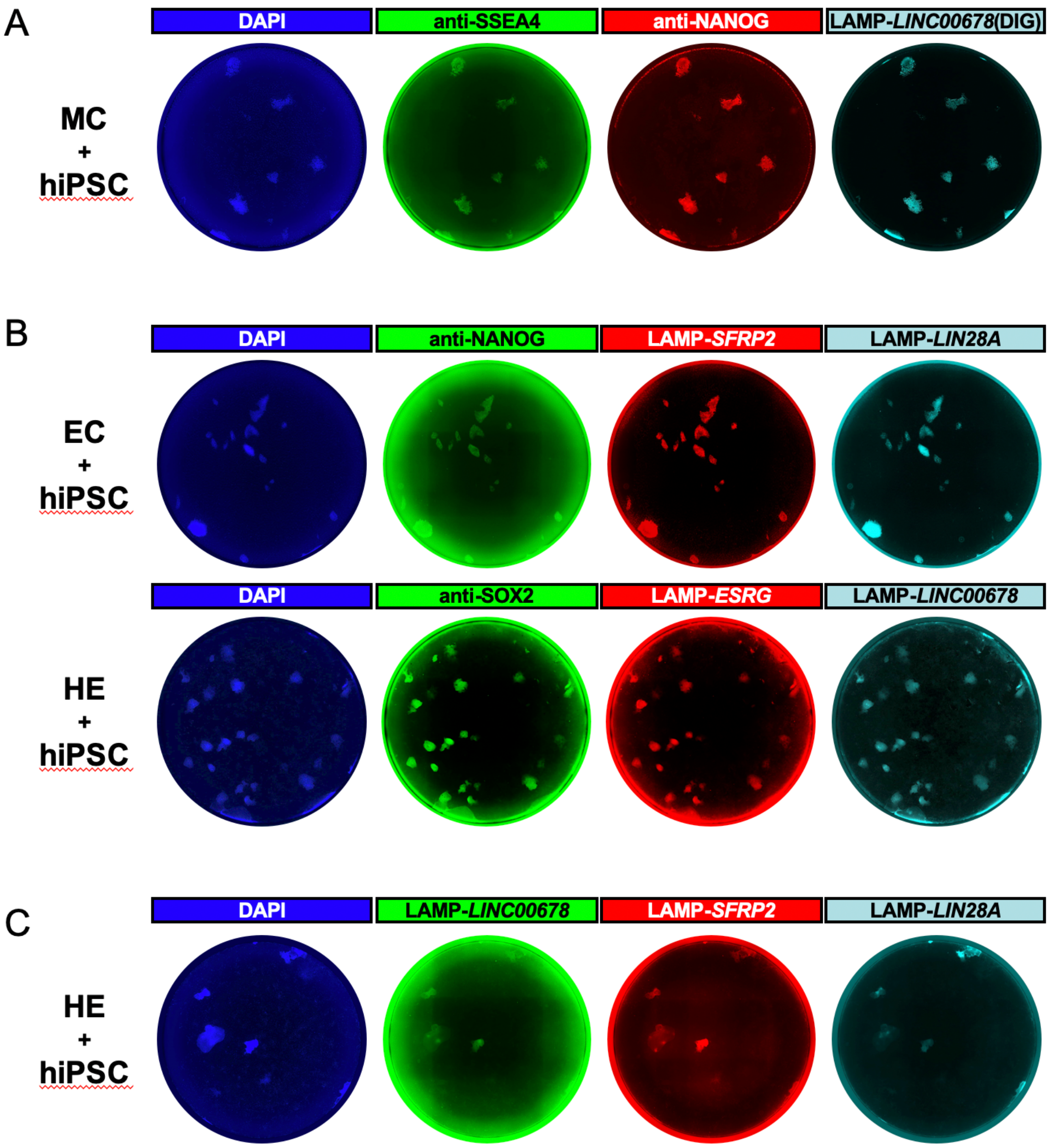

Supplementary Figure 10. Specific detection of undifferentiated cells by *in situ* RT-LAMP. hiPSC colony detection by combinations of immunostaining and *in situ* RT-LAMP in the culture assay process staining DIG-labeled (A), double (B), or triple (C) fluorolabelled target genes. Images of whole wells (well bottom diameter, 6.35 mm).

Supplementary Table 1. qPCR primers and probes.

GENES			SEQUENCE 5' > 3'
ACTB	PRIMERS	FORWARD	ATTGGCAATGAGCGGTTC
		BACKWARD	GGATGCCACAGGACTCCAT
	PROBE		UPL No.11
B2M	PRIMERS	FORWARD	TTCTGGCCTGGAGGCTATC
		BACKWARD	TCAGGAAATTTGACTTTCCATTC
	PROBE		UPL No.42
CNMD	PRIMERS	FORWARD	ACTCACAAGCCTTCAATCCTG
		BACKWARD	TCTAGGGTCGAATGTCATGCT
	PROBE		UPL No.18
GAPDH	PRIMERS	FORWARD	AGCCACATCGCTCAGACAC
		BACKWARD	GCCCAATACGACCAAATCC
	PROBE		UPL No.60
ESRG	PRIMERS	FORWARD	TGGGTCTTTCAAGAAGTTCCTC
		BACKWARD	TGGGATGGAGCCATAGAAGT
	PROBE		UPL No.52
LIN28A	PRIMERS	FORWARD	GAAGCGCAGATCAAAAGGAG
		BACKWARD	GCTGATGCTCTGGCAGAAGT
	PROBE		UPL No.23
LINC00678	PRIMERS	FORWARD	CATCTCACCAATTTTAAATCAGGAC
		BACKWARD	CTCCCGTCATTCTGCTAACAC
	PROBE		UPL No.17
NANOG	PRIMERS	FORWARD	ATGCCTCACACGGAGACTGT
		BACKWARD	CAGGGCTGTCCTGAATAAGC
	PROBE		UPL No.69
OCT4	PRIMERS	FORWARD	CTTCGCAAGCCCTCATTTC
		BACKWARD	GAGAAGGCGAAATCCGAAG
	PROBE		UPL No.60
PRDM14	PRIMERS	FORWARD	TGCACCATGCGATTTCAG
		BACKWARD	TGCATGAGGCATAGACCTTC
	PROBE		UPL No.79
SFRP2	PRIMERS	FORWARD	GCTAGCAGCGACCACCTC
		BACKWARD	TTTTTGCAGGCTTCACATACC
	PROBE		UPL No.83
SOX2	PRIMERS	FORWARD	GGGGGAATGGACCTTGTATG
		BACKWARD	GCAAAGCTCCTACCGTACCA
	PROBE		UPL No.65
TNFRSF8	PRIMERS	FORWARD	GCTGTCAGGAGGTGCTGTTAC
		BACKWARD	GTAGGCCTCTGTGGGCACT
	PROBE		UPL No.43
USP44	PRIMERS	FORWARD	TGATGGAAACTGGGCGATCCTGC
		BACKWARD	TGAGGGTTGAGGCTGGAATGGTC
	PROBE		UPL No.44

Supplementary Table 2. LAMP primers and probes.

Gene / Refseq Accession number			SEQUENCE 5'>3'
ESRG	PRIMERS	FOP	GGAACTCTGGCCCAAGGT
NR_027122		BOP	CTGTGTGAAGAGACCACCAA
		FIP	TTCTGAGGCGATCAGGCAGCCTCCTTCTTGGCTTACTGGC
		BIP	GCAGACCATCATGGACGCCGAGGCTTTGTGTGAGCAACA
		BLP	GCTTTAGCCCGCCTGCA
		Labelled BLP	Alexa Fluor 555 - GCTTTAGCCCGCCTGCA
	PROBE		GCCTGCACCCAGGTGAAATAAACAGCC - BODIPY FL
			GCCTGCACCCAGGTGAAATAAACAGCC - TAMRA
LIN28A	PRIMERS	FOP	TTCCTGTCCATGACCGC
NM_024674.6		BOP	TCCTTTTGGCCGCCTCT
		FIP	TCCGGAACCCTTCCATGTGCAGCGACCCCCCAGTGGATGTC
		BIP	AGTTCACCTTTAAGAAGTCAGCCACACTCCCAATACAGAATACTCC
		BLP	AGGGTCTGGAATCCATCCG
		Labelled BLP	Cy5 - AGGGTCTGGAATCCATCCG
	PROBE		GGAATCCATCCGTGTCACCGGACC - BODIPY FL
LINC00678	PRIMERS	FOP	CCCTGTCCTCCTGTTCTT
NR_102708.1		BOP	CAGGAGCTGATTCTGTTGC
		FIP	GTTCGTTGGGCTGGTCGGCTCCGTGAGAAAGATCCACCTA
		BIP	AAATCAGGACCTACCAGTCTGCCCGTCTCGCAGGGTATCAGTC
		BLP	TGCTCATACTTGATCTGGATGA
		Labelled BLP	Cy5 - TGCTCATACTTGATCTGGATGA
		Labelled BLP	ATTO 488 - TGCTCATACTTGATCTGGATGA
	PROBE		AGGTCCTCAGACCGACCAGCCC - BODIPY FL
PRDM14	PRIMERS	FOP	TCGGTTCCAGTTCACGG
NM_024504.4		BOP	GACTTCACCAAACACCGTC
		FIP	ATGGTGCAGGCTGGCTGGGGGAGGACCTGCACTTCGTT
		BIP	TCCCCCCAGACAGCTCTGGCATAGACCTTCTGGAAGTTGAA
		FLP	TGCTCCAGGCTGGGAGTGAC
		BLP	ATCTGATTCTCTTCCTCAAACTCTG
	PROBE		TTCTCTTCCTCAAACTCTGGATAAAGACTCCC-BODIPY FL
SFRP2	PRIMERS	FOP	TCCAAAGGTATGTGAAGCCT
NM_003013.3		BOP	TCATCTCCTCACAGGTGC
		FIP	TCTCGGTTGATGTAGGTTATCTCCTTGACAACGACATAATGGAAACGC
		BIP	TGGAGACCAAGAGCAAGACCATTTGTCTTTGAGCCACAGCAC
		BLP	TTTACAAGCTGAACGGTGTGTC
		Labelled BLP	Alexa Fluor 555 - TTTACAAGCTGAACGGTGTGTC
	PROBE		GGTGTGTCCGAAAGGGACCTGAAGAAATC - BODIPY FL
USP44	PRIMERS	FOP	CCGCCAGGACTTTTCACT
NM_032147.5		BOP	AGCTGAGAAATGCATAATCCAA
		FIP	TAACTCAGAGGTCAGTCCCTGTAGGAGATCAGCATTTGCCCTG
		BIP	CCAAAGGCCGACCTGGGGAAAGTCTCTGTTCACAACACTTG
		FLP	GGATCGCCCAGTTTCCAT
	PROBE		BODIPY FL-CTGATTTTGAGGTTTTAATAGTTTTCAGATGCTT

Supplementary Table 3. RT-LAMP reagents.

Reagent	*In vitro* assay
Tricine	10 mM
$MgSO_4$	8 mM
KCl	30 mM
Dextran	1.5%
Fish collagen peptides	1.0%
Dithiothreitol	1 mM
Tween 20	0.2%
FOP	0.2 μM
BOP	0.2 μM
FIP	1.6 μM
BIP	1.6 μM
LFP (For *PRDM14* and *USP44*)	0.8 μM
LBP	0.8 μM
Probe	40 nM
SYTO63	188 nM

Supplementary Table 4. *in vitro* RT-LAMP conditions.

Related Figs	Target gene	Tested cell or model	Tested nucleic acid (µg/test)	dNTPs (mM)	*Bst* polymerase (U/µL)	Reverse Transcriptase (U/µL)	LAMP temperature (℃)
Fig. 1D and 1E	*ESRG*	ssssDNA + artificial DNA	0 - 10	1.4	0.36	0	63
Fig. 1D and 1E	*SFRP2*	ssssDNA + artificial DNA	0 - 10	1.4	0.36	0	63
Fig. 1F	*SFRP2*	ssssDNA + artificial DNA	0 - 100	1.4	0.36	0	63
Fig. 2	*ESRG*	HE	1	0.9	0.36	0.06	63
Fig. 2	*LINC00678*	HE	1	1.4	0.12	0.12	63
Fig. 2	*PRDM14*	HE	1	0.6	0.36	0.06	63
Fig. 3A and S6	*ESRG*	EC/MC/NPC/293T	5	0.9	0.36	0.06	63
Fig. 3A and S6	*LINC00678*	HE/EC/293T	5	1.4	0.12	0.12	67
Fig. 3A and S6	*USP44*	EC/HeLa	5	0.9	0.36	0.06	63
Fig. 3B and S7	*ESRG* and *LINC00678*	EC	5	1.1	0.36	0.06	67
Fig. S5A	*LINC00678*	Artificial DNA	0	0.4 - 1.4	0.36	0.06	63
Fig. S5B	*LINC00678*	HE ± Artificial DNA	1	0.7 - 1.3	0.36	0.06	63
Fig. S6	*LIN28A*	HeLa	5	1.4	0.12	0.12	63
Fig. S6	*SFRP2*	293T	5	1.4	0.12	0.12	65

Supplementary Table 5. Antibodies.

Antibody	Target protein	Host and isotype	Label	Company	Catalog No.	Dilution rate
Primary antibody	SOX2	Rabbit monoclonal IgG	-	Cell Signaling Technology	9656S	1/200
	OCT4A	Rabbit monoclonal IgG	-	Cell Signaling Technology	9656S	1/200
	NANOG	Rabbit monoclonal IgG	-	Cell Signaling Technology	9656S	1/200
	TRA1-81	Mouse monoclonal IgM	-	Cell Signaling Technology	9656S	1/200
	SSEA4	Mouse monoclonal IgG3	-	Cell Signaling Technology	9656S	1/200
	Digoxigenin	Mouse monoclonal IgG1κ	-	Sigma-Aldrich	11333062910	1/100
Secondary antibody	Rabbit IgG	Donkey polyclonal IgG	Alexa Fluor 488	Thermo Fisher Scientific	A21206	1/1000
	Rabbit IgG	Donkey polyclonal IgG	Alexa Fluor 555	Thermo Fisher Scientific	A31572	1/1000
	Mouse IgM	Goat polyclonal IgG	Alexa Fluor 488	Thermo Fisher Scientific	A21042	1/1000
	Mouse IgG3	Goat polyclonal IgG	Alexa Fluor 488	Thermo Fisher Scientific	A21151	1/1000
	Mouse IgG1	Goat polyclonal IgG	Alexa Fluor 647	Thermo Fisher Scientific	A21240	1/1000

Supplementary Table 6. *In situ* RT-LAMP conditions.

Target genes	Reagent	DIG labelling	Double fluoro-labelling	Triple fluoro-labelling
	Tricine	10 mM	10 mM	10 mM
	MgSO4	8 mM	8 mM	8 mM
	KCl	30 mM	30 mM	30 mM
	Dextran	1.5%	1.5%	1.5%
	Fish collagen peptides	1.0%	1.0%	1.0%
	Dithiothreitol	1 mM	1 mM	1 mM
	Tween 20	0.2%	0.2%	0.2%
	Digoxigenin-11-dUTP	40 μM	-	-
	WarmStart RTx Reverse Transcriptase	0.06 U/μL	0.06 U/μL	0.06 U/μL
	Bst 2.0 WarmStart DNA Polymerase	0.36 U/μL	0.36 U/μL	0.72 U/μL
Gene 1	FOP	0.2 μM	0.2 μM	0.05 μM
LIN28A or *LINC00678*	BOP	0.2 μM	0.2 μM	0.05 μM
	FIP	1.6 μM	1.6 μM	0.4 μM
	BIP	1.6 μM	1.6 μM	0.4 μM
	Non- or Fluoro-labelled BLP	0.8 μM	0.8 μM	0.6 μM
Gene 2	FOP	-	0.2 μM	0.05 μM
SFRP2 or *ESRG*	BOP	-	0.2 μM	0.05 μM
	FIP	-	1.6 μM	0.4 μM
	BIP	-	1.6 μM	0.4 μM
	Fluoro-labelled BLP	-	0.8 μM	0.6 μM
Gene 3	FOP	-	-	0.8 μM
LINC00678	BOP	-	-	0.8 μM
	FIP	-	-	6.4 μM
	BIP	-	-	6.4 μM
	Fluoro-labelled BLP	-	-	4.8 μM

Supplementary Table 7. Residual undifferentiated cells among normal HE or over-passed hiPSC derived HE.

	Colony number	Population of undifferentiated cell
Normal HE	0	0.000%
	0	0.000%
	0	0.000%
Over-passed hiPSC derived HE	2.5	0.002%
	2.5	0.002%
	4.5	0.003%
	6	0.004%
	7	0.004%
	19	0.012%
	53	0.033%
	62.5	0.039%
	77	0.048%
	86	0.054%
	87.5	0.055%
	99	0.062%
	132.5	0.083%
	155	0.097%
	189.5	0.118%
	391.5	0.245%
	659	0.412%
	665.5	0.416%

Observed residual undifferentiated cell colony numbers among normal HE (N = 3 biological replicates, N = 2 replicate wells, mean) or over-passed hiPSC derived HE (N = 18 biological replicates, N = 2 replicate wells, mean). Populations of undifferentiated cells were calculated by dividing the colony numbers by the seeded cell number, 1.6×10^5.
